# Postural adaptations to unilateral knee joint hypomobility induced by orthosis wear during gait initiation

**DOI:** 10.1038/s41598-018-19151-1

**Published:** 2018-01-16

**Authors:** A. Delafontaine, P. Fourcade, J. L. Honeine, S. Ditcharles, E. Yiou

**Affiliations:** 1 CIAMS, Univ. Paris-Sud., Université Paris-Saclay, 91405 Orsay, France; 2 CIAMS, Université d’Orléans, 45067 Orléans, France; 3 CSAM Laboratory, Department of Public Health, University of Pavia, Pavia, Italy

## Abstract

Balance control and whole-body progression during gait initiation (GI) involve knee-joint mobility. Single knee-joint hypomobility often occurs with aging, orthopedics or neurological conditions. The goal of the present study was to investigate the capacity of the CNS to adapt GI organization to single knee-joint hypomobility induced by the wear of an orthosis. Twenty-seven healthy adults performed a GI series on a force-plate in the following conditions: without orthosis (“control”), with knee orthosis over the swing leg (“orth-swing”) and with the orthosis over the contralateral stance leg (“orth-stance”). In orth-swing, amplitude of mediolateral anticipatory postural adjustments (APAs) and step width were larger, execution phase duration longer, and anteroposterior APAs smaller than in control. In orth-stance, mediolateral APAs duration was longer, step width larger, and amplitude of anteroposterior APAs smaller than in control. Consequently, step length and progression velocity (which relate to the “motor performance”) were reduced whereas stability was enhanced compared to control. Vertical force impact at foot-contact did not change across conditions, despite a smaller step length in orthosis conditions compared to control. These results show that the application of a local mechanical constraint induced profound changes in the global GI organization, altering motor performance but ensuring greater stability.

## Introduction

Gait initiation (GI) is the transient phase between quiet standing posture and ongoing walking. It is a functional task that is classically used in the literature to investigate the coordination between posture and movement^1–8^. It is composed of a postural phase preceding swing foot-off, which corresponds to the “Anticipatory Postural Adjustments” (APAs), followed by a step-execution phase ending at the time of swing foot-contact with the ground. APAs are known to be predictive of GI in both motor performance^1,9^ and postural stability^5,6,10–12^. Along the progression axis, APAs consist of a backward center of pressure (COP) displacement which generates the forward propulsive forces required to reach the intended motor performance, in terms of step length and progression velocity^1,9^. Along the mediolateral axis, APAs involve shifting the COP toward the swing leg, which promotes center of mass (COM) displacement in the direction of the stance leg^5,6,8^. Mediolateral APAs reduce the distance between the COM and the COP at foot-off in the frontal plane. This acts to attenuate the mediolateral COM fall toward the swing leg during the execution phase that is caused by gravity^5,6,11–13^. Anticipatory backward COP shift is due to bilateral dorsi-flexion of the ankles^8,14,15^ while anticipatory mediolateral COP shift toward the swing leg is classically attributed to ipsilateral hip adduction^16^. Ankle and hip mobility during APAs are therefore crucial to ensure postural stability and forward body propulsion. Consistently, recent studies have showed that blocking unilaterally or bilaterally the ankle(s) with strapping induces an alteration in COP backward shift and thus a degradation of motor performance^17^.

To date, the role of knee mobility in the biomechanical organization of GI has received far less attention. Honeine *et al*.^8^ reported that stance knee flexion during APAs, combined with ipsilateral hip adduction^16^, would contribute to the anticipatory mediolateral COP shift and would thus be involved in the maintenance of postural stability. In the same vein, Yiou *et al*.^5^ recently reported that the mediolateral stance leg stiffness (or “stance leg stiffness” for text simplification thereafter) during GI execution was an important parameter that could influence postural stability at swing foot-contact. In this study, the stance leg stiffness was computed along the mediolateral direction as follow: k = T/yCOM, where T, corresponded to an elastic restoring force that reflected the lateral control of the COM motion exerted by stance leg muscles, and yCOM corresponded to the COM shift, which was systematically oriented towards the swing leg during the GI execution phase. The model predicted that an increase in the stance leg stiffness during the execution phase may increase the lateral COM fall. The extent to which the stance knee mobility during the execution phase influences k and thus stability, is to date unknown.

Swing knee mobility is also involved in the maintenance of postural stability, with knee flexion at the end of APAs contributing to the clearing of the swing foot from the ground and thus avoid stumbling. Swing knee mobility is also involved in GI performance, with knee extension during GI execution contributing to reach the intended step length^18–20^. In addition, the collision of the swing foot with the support surface is known to generate a peak vertical force impact, the amplitude of which depends on step length/progression velocity^21,22^, and on the capacity of the stance leg to brake the vertical COM fall during the execution phase^2,8^. The flexion of the swing knee after foot-contact is also important to attenuate the transmission of these disturbing forces to the hip and upper body, and thus avoid discomfort or pain with task repetition^23–25^. Any restriction of unilateral knee mobility or default in knee control may thus potentially affect postural stability, motor performance and cause discomfort or pain during GI – unless participants are able to develop adaptive strategies that remain to be explored.

Restriction of knee mobility due to orthopedic impairments is commonly found in elderly^18^, patients with knee arthritis^20^ or wearing medical devices such as an orthosis; the most extreme case being that of upper knee amputees. In this latter population, the strategy to control the forward propulsive forces to reach the intended motor performance has been shown to change in an adaptive manner according to the limb (sound or prosthetic) that initiated gait^19^. Motor performance however remained weaker in this population as compared to able-body participants^26^. It is noteworthy that the question whether unilateral knee immobility influenced mediolateral APAs, leg stiffness and the related postural stability was not investigated in these studies. Nonetheless, recent studies reported that the central nervous system (CNS) in able-bodied participants was able to adapt mediolateral spatiotemporal features of APAs to various constraints imposed on the postural system, such as obstacles of changing height and distance^5^, temporal pressure^5,6^, fear of falling^27,28^, speed instruction^29^, asymmetrical body loading^30^, and initial stance width^8^ in order to maintain stability.

Beside knee hypomobility due to orthopedic impairments, default in knee control is often reported in neurological patients, e.g. in patients suffering from stroke^31^, Parkinson’s disease^32,33^, cerebral palsy^34^, or multiple sclerosis^35^. Knee pain may also induce protective changes in motor control during locomotor activities^36^. Interestingly, freezing of gait in Parkinsonian patients is associated with knee trembling^37–42^. Jacobs *et al*.^43^ found that during GI, knee trembling caused right-left leg loading-unloading cycles. According to Honeine *et al*.^8^, knee flexion caused unloading in the flexed limb resulting in displacing the COP in the opposite direction which could in turn explain the multiple APAs that are observable during freezing. In addition, smaller mediolateral COP shift during APAs with longer duration and larger step width of the first step of GI have been observed in Parkinson’s disease^32,33^. Enlarging the step width during gait initiation is a strategy that is used by the CNS to ensure stability in case the amplitude of mediolateral APAs is not sufficient^11,12,29,44^. Now, whether the changes in the GI biomechanical organization reported in the above-mentioned studies are primarily a response to normal adaptation to knee hypomobility; or rather to abnormal APA programming linked to impairment in the neural pathways/structures involved in the coordination between posture and movement, remains unclear in current literature.

The goal of the present study was to investigate the capacity of the CNS to adapt the GI organization to a mechanical perturbation applied to a single component of the locomotor system, namely the knee joint component. Knee joint was specifically targeted by the perturbation because of its key role in the maintenance of postural stability during GI, as underlined above. The perturbation was induced by means of an orthosis placed over the stance- or swing-leg knee. This device restricted joint mobility, i.e. it induced knee hypomobility. Because the role of each leg during GI is asymmetrical (one leg is involved in stepping, one leg is involved in stance), unilateral rather than bilateral perturbation was induced so as to better highlight adaptations that are specific to stance and swing knee hypomobility. It is also noteworthy that unilateral leg impairments due to knee hypomobility are most commonly encountered than bilateral impairments in traumatology, neurology, rheumatology and in post-surgery conditions. It is hypothesized that stance- and swing-leg knee hypomobility will require specific adaptive changes in the GI stabilizing features (including mediolateral APAs features, stance leg stiffness and step width) so as to maintain stability unchanged, but will induce a degradation of motor performance as compared to GI without orthosis.

## Results

### Biomechanical traces

**Figure 1:**
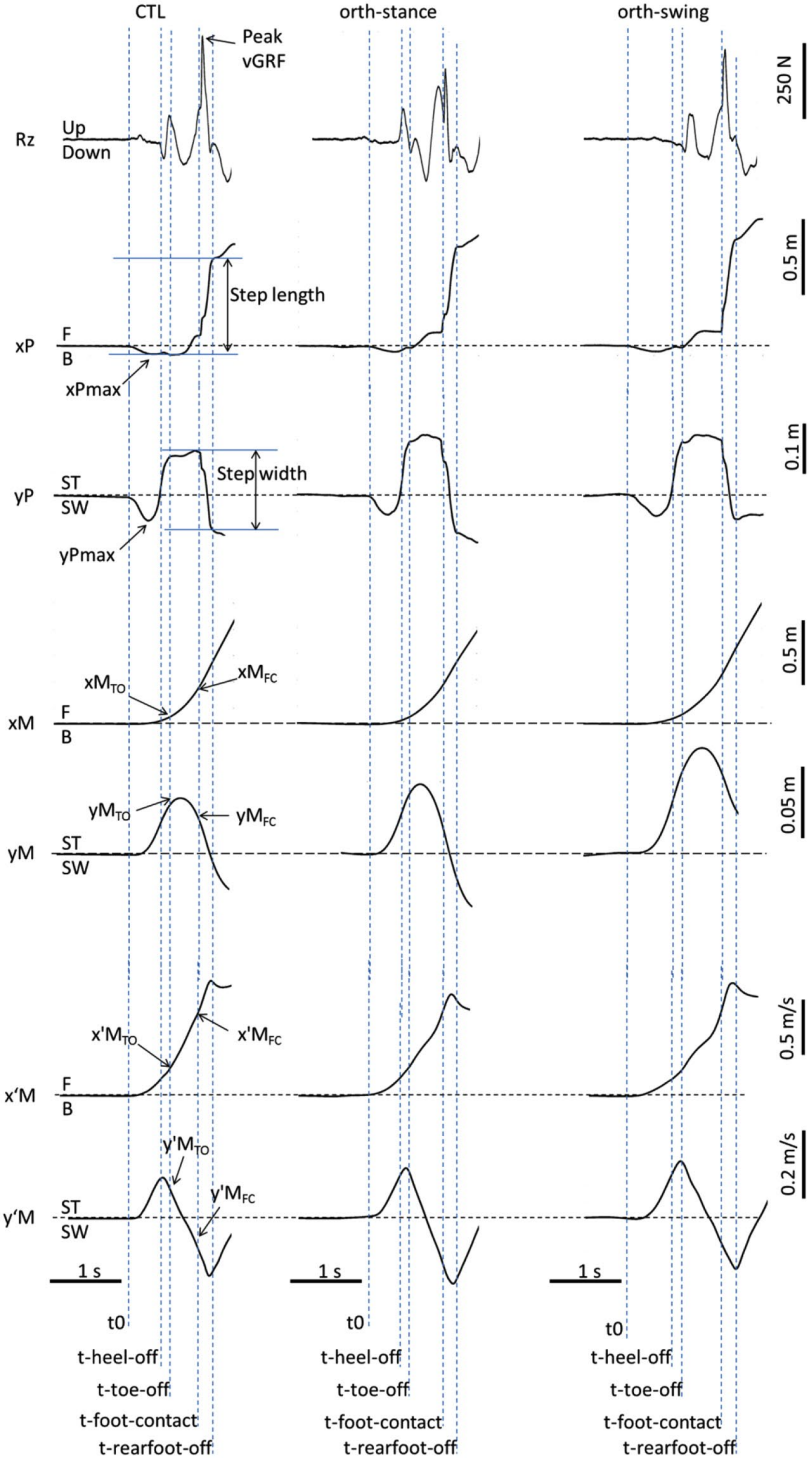
Typical biomechanical traces in the three experimental conditions of the main experiment (one representative participant). Rz is the vertical ground reaction force. xCOP, xCOM and x’COM are respectively the center of pressure (COP) displacement, the COM displacement and COM velocity along the x axis which corresponded to the anteroposterior axis. xCOP_MAX_, xCOM_TO_, xCOM_FC_ are respectively the peak of the COP position reached during APAs, the COM position at toe off (TO) and the COM position at foot contact (FC). yCOP, yCOM and y’COM are respectively the COP displacement, the COM displacement and the COM velocity along the y axis which corresponded to the mediolateral axis. B and F are respectively the backward and forward directions. SW and ST are respectively the swing and stance limbs. t0, t-heel-off, t-toe-off, t-foot-contact and t-rear foot-off are respectively the instants of gait initiation onset, swing heel-off, swing toe-off, swing foot-contact and rear foot-off.

In each condition, swing heel-off was systematically preceded by dynamic phenomena that corresponded to APAs (Fig. 1). During these APAs, the COP displacement peaked in the backward direction and towards the swing leg, while the COM velocity was directed forward and towards the stance leg. During the execution phase, the mediolateral COP trace reached a first “quasi-plateau”, while the anteroposterior COP was progressively shifted toward the toes. At swing foot-contact, the COP was shifted forward and towards the swing leg, and the peak vertical Ground Reaction Force at foot contact (or “peak Rz”, Rz being the vertical ground reaction force) trace increased sharply to reach a peak. The mediolateral COP trace reached a second “quasi-plateau” at rear swing foot-off. The mediolateral COM velocity trace peaked towards the stance leg at around heel-off then peaked towards the swing leg at around swing foot-contact. The anteroposterior COM velocity peaked following foot-contact. Finally, visual observation of the participants’ behavior showed an outward rigid swing leg motion during the execution phase in the orth-swing condition, which was not the case in the two other conditions.

### Main experiment

#### COP position in the initial posture

Results showed that there was no significant effect of the condition on the initial COP position along the anteroposterior and the mediolateral axis.

### APAs and foot-lift phase

#### Mediolateral direction

**Figure 2:**
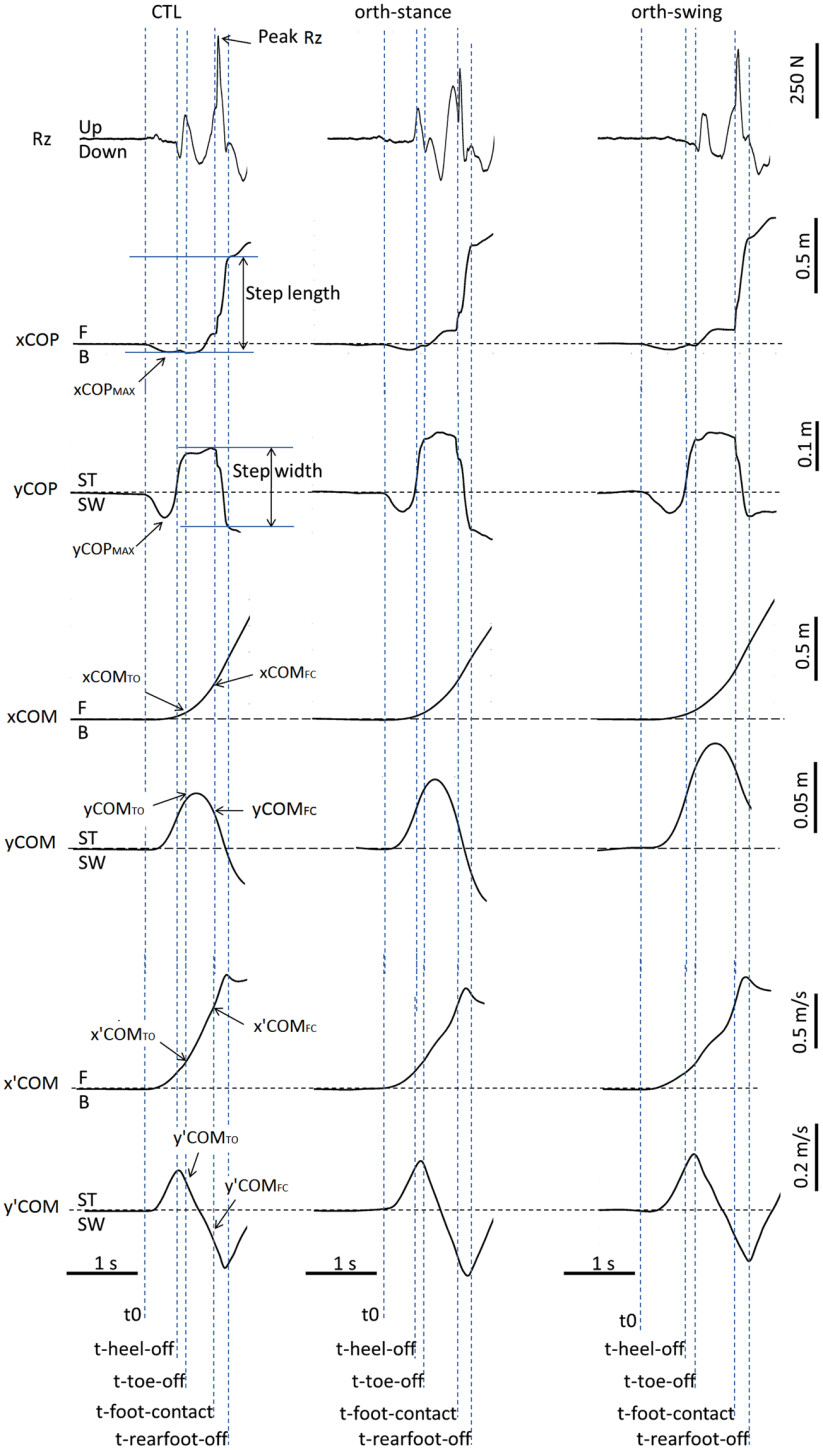
Comparison of selected APAs parameters between the three conditions in the main experiment. Reported are mean values (all participants together) ±1 SD. *, ** and *** indicates a significant difference between bars with respectively p < 0.05, p < 0.01 and p < 0.001.

There was a significant effect of the condition on the following variables: peak of anticipatory COP shift (*F*_(2,34)_ = 9.52, *p* < 0.001), COM velocity at toe-off (*F*_(2,34)_ = 7.75, *p* < 0.01) and APAs duration (*F*_(2,34)_ = 5.04, *p* < 0.05). *Post hoc* analysis further revealed that the two former variables were significantly higher in the orth-swing condition than in the control and the orth-stance condition. APAs duration along the mediolateral direction was longer in the orth-stance than in both the control and the orth-swing condition (Fig. 2 for details of the *post hoc* analysis).

#### Anteroposterior direction

There was a significant effect of the condition on the peak of anticipatory backward COP shift (*F*_(2,34)_ = 26.93, *p* < 0.001) and on the forward COM velocity at toe-off (*F*_(2,34)_ = 67.18, *p* < 0.001). *Post hoc* analysis further revealed that these two variables significantly decreased from the control, to the orth-swing and the orth-stance condition (Fig. 2e,f). In contrast, there was no significant effect of the condition on the duration of APAs and foot-lift phases (Fig. 2d,f).

### Execution phase

**Figure 3:**
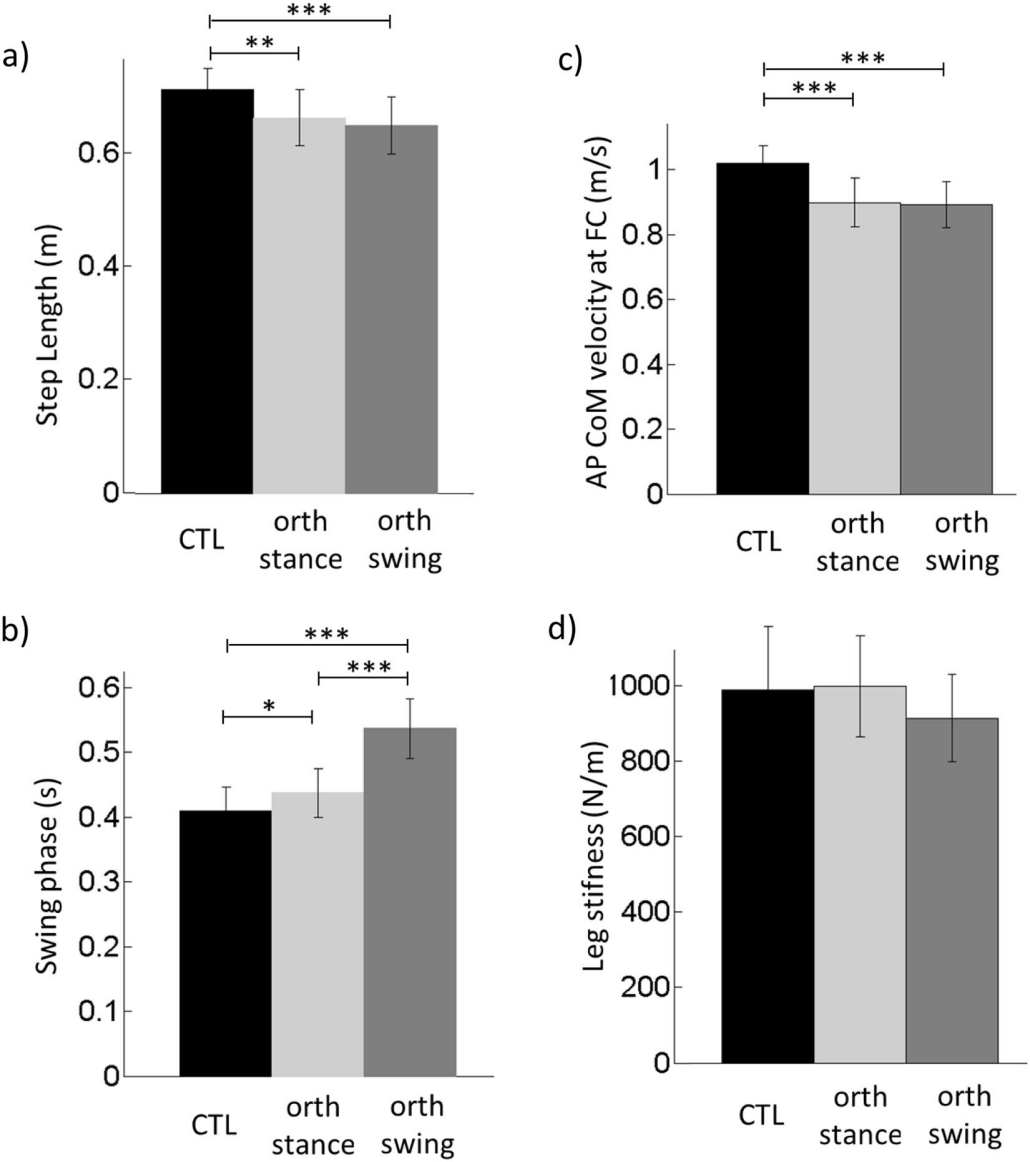
Comparison of execution phase related parameters between the three conditions in the main experiment. Reported are mean values (all participants together) ±1 SD. *, ** and *** indicates a significant difference between bars with respectively p < 0.05, p < 0.01 and p < 0.001.

There was a significant effect of the condition on the following variables: execution phase duration (*F*_(2,34)_ = 71.87, *p* < 0.001), step length (*F*_(2,34)_ = 13.18, *p* < 0.001) and progression velocity (*F*_(2,34)_ = 24.72, *p* < 0.001). *Post hoc* analysis revealed that the swing (i.e. execution) phase duration was the longest in the orth-swing condition and the shortest in the control condition (Fig. 3b). The step length and the progression velocity were both significantly higher in the control than in the two orthosis conditions (Fig. 3a,c). In contrast, there was no significant effect of the condition on stance leg stiffness (Fig. 3d). The best fitting shape between the experimental and the theoretical traces of the mediolateral COM velocity and position was obtained for a leg stiffness of 966 ± 139 N/m (mean value all conditions pooled together).

### Stability

**Figure 4:**
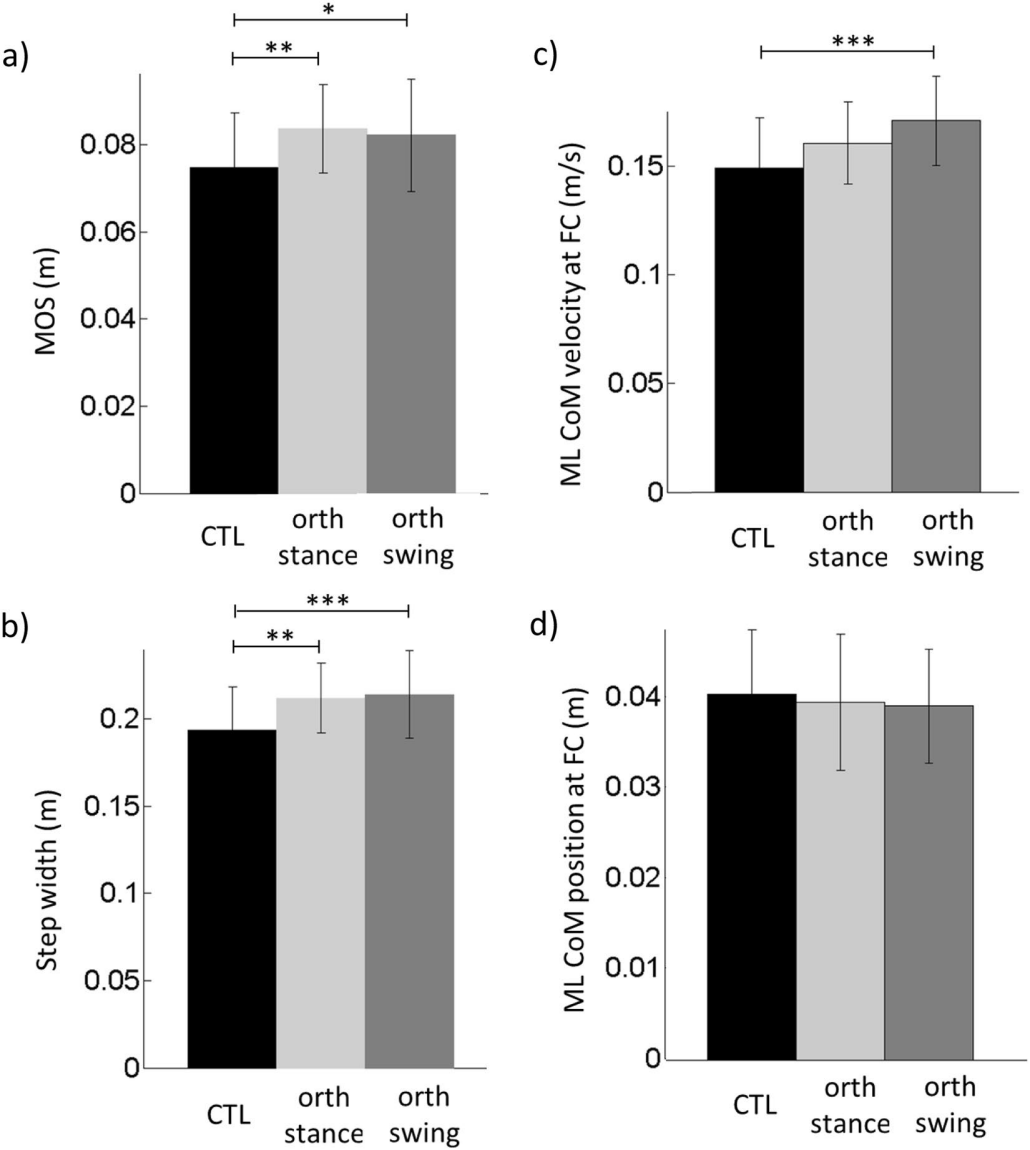
Comparison of stability related parameters between the three conditions in the main experiment. Reported are mean values (all participants together) ±1 SD. *, ** and *** indicates a significant difference between bars with respectively p < 0.05, p < 0.01 and p < 0.001. MOS is the Margin Of Stability.

There was a significant effect of the condition on the following variables: step width (*F*_(2,34)_ = 11.45, *p* < 0.001), mediolateral COM velocity measured at swing foot-contact (*F*_(2,34)_ = 8.9, *p* < 0.001) and MOS (*F*_(2,34)_ = 6.11, *p* < 0.01). In contrast, the condition had no effect on the mediolateral COM position at swing foot-contact (Fig. 4d). *Post hoc* analysis showed that the mediolateral COM velocity at swing foot-contact was significantly larger in the control than in the orth-swing condition (Fig. 4c), but it was not significantly different between the control and the orth-stance condition (it did, however, tend to be larger in the control condition, with p < 0.08); nor was it different between the two orthosis conditions. The step width and the MOS were both significantly larger in the two orthosis conditions than in the control condition (Fig. 4a,b).

### Vertical force impact and vertical COM braking

There was no significant effect of the condition on the peak Rz value, the associated slope and the vertical braking index.

### Complementary experiment

**Figure 5:**
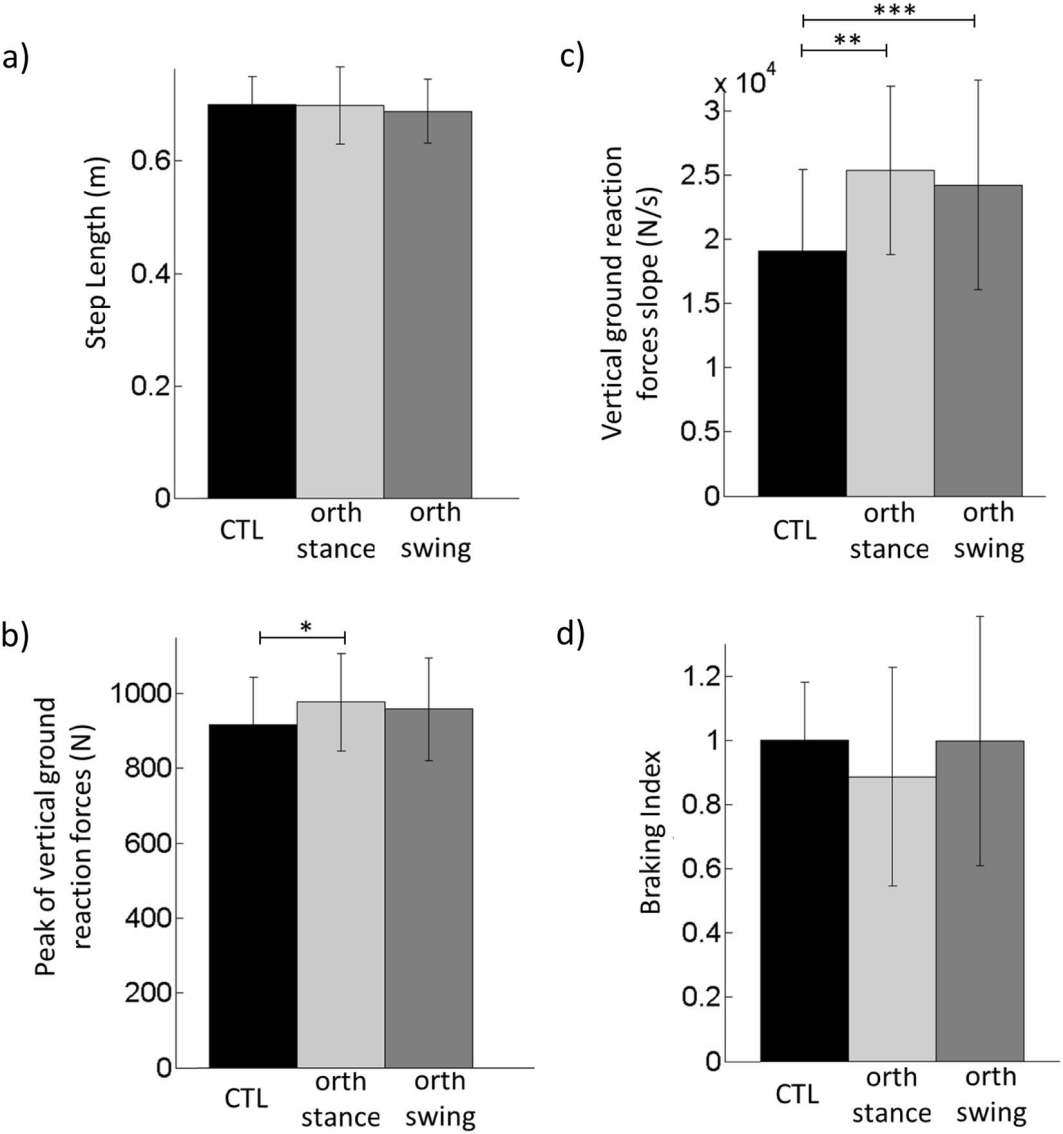
Comparison of step length and vertical force impact related variables between the three conditions in the complementary experiment. Reported are mean values (all participants together) ±1 SD. *, ** and *** indicates a significant difference between bars with respectively p < 0.05, p < 0.01 and p < 0.001.

There was no significant effect of the condition on the step length (Fig. 5a). Participants in the two orthosis conditions were therefore able to reach the same step length as in the control condition when instructed to do so. Conversely, the condition did have a significant effect on peak Rz value and on its associated slope (*F*_(2,14)_ = 4.56, *p* < 0.05) and on its associated slope (*F*_(2,34)_ = 9.14, *p* < 0.01). *Post hoc* analysis showed that the peak Rz value was significantly larger in the orth-stance than in the control condition (Fig. 5b) and that the associated slope was significantly larger in the two orthosis conditions than in the control condition, with no difference between the two orthosis conditions (Fig. 5c). Finally, there was no significant effect of the condition on the braking index (Fig. 5d).

## Discussion

This study investigated the effect of unilateral knee hypomobility on the GI biomechanical organization. Knee hypomobility was experimentally induced by means of an orthosis placed unilaterally over the stance- or swing-leg knee. It was hypothesized that unilateral stance- and swing-leg knee hypomobility will require adaptive changes in the GI stabilizing features for stability to remain unchanged, but will induce a degradation of the motor performance as compared to GI without orthosis. Results of the present study corroborate these hypotheses and suggest that restricting lower limb joint mobility unilaterally induces profound modifications in the GI biomechanical organization.

Results showed that swing knee hypomobility induced a large reduction in the amplitude of the mediolateral APAs, in terms of peak COP shift. As the duration of these APAs did not change in comparison with the control condition, it resulted that the initial (foot-off) velocity of the COM was increased. This change in the mediolateral APAs amplitude could *a priori* be surprising since recent study by Honeine *et al*.^8^ reported that the swing knee began to flex only *after* the peak of COP shift was reached, i.e. that knee mobility could not be involved in the mediolateral APAs. This change in APAs amplitude can be ascribed to the large increase in the swing phase duration (24%) in the orth-swing condition compared to the control condition. Previous studies indeed showed that the longer this duration, the larger the mediolateral fall of the COM at the time of swing foot-contact, with potential risks of imbalance towards the swing leg^5,6,10–12^. The main function of these mediolateral APAs is to maintain this lateral fall within limits compatible with stability maintenance at foot-contact^5^. Note however that the mediolateral COM fall at this time (in terms of COM velocity) was larger in the orth-swing condition compared to the control condition, which suggests that the increase in APAs amplitude was not fully sufficient to compensate for the swing phase duration increase. This increase swing duration was neither compensated by a change in the stance leg stiffness. Our mechanical model indeed predicted that a decrease in the mediolateral stance leg stiffness during the execution phase may have reduced this lateral fall. Leg stiffness however remained the same across the three conditions, suggesting that knee mobility was not involved in leg stiffness, at least along the mediolateral axis (the stance leg stiffness was solely computed along this axis). It is possible that the mediolateral leg stiffness might merely be dependent on the stance hip and/or ankle mobility, which were not affected by the stance knee orthosis. On this aspect, it should be stressed that, in contrast to stance-leg hip and ankle, the body cannot pivot laterally around the stance knee during GI, simply because of joint anatomy. Adding an orthosis at this joint could thus not have affected stance leg stiffness in the frontal plane since this joint has no mobility in this plane. Future study will investigate this hypothesis.

The step width increase observed in the orth-swing condition might be the result of modified hip and ankle strategies directed to compensate for the increased execution phase duration. Unfortunately, we did not record body kinematics data and we cannot therefore properly quantify these complementary strategies. Nonetheless, a similar finding was reported during compensatory stepping initiation over an obstacle in reaction to sudden support surface translation^12^ or during rapid compared to spontaneous GI^29^. In the present study, the large increase in the execution phase duration can be ascribed to the observed outward rigid movement of the swing leg during the execution phase. This mowing leg movement, although not measured in our study is well-known in stroke patients with stiff knee gait^31,45^ and in patients wearing knee orthosis^46^, and it was systematically observed in all participants in the orth-swing condition only. This strategy serves to clear the swing foot from the ground in a condition where the swing knee is blocked^45^. It also serves to shift the swing leg forward while avoiding friction between the foot and the support surface, and so reduces the risk of stumbling. Clearly, this compensatory strategy increased the duration of the execution phase compared to moving the leg straightforward, as it was observed in both the orth-stance and the control conditions.

Results further showed that the progression velocity and the step length were both drastically reduced in the orth-swing condition (and the orth-stance condition) compared to the control condition. Recalling the action of the swing knee during GI in normal condition may help to better understand this negative effect. As stated above, the swing knee flexion facilitates foot clearance during normal GI. This knee flexion reaches a peak (~45°) during the execution phase. The knee then extends to reach the desired step length. After foot-contact, the swing knee flexes again so as to attenuate the transmission of the vertical force impact from the swing foot to the hip and the above structures^23–25^. The small flexion of the stance knee throughout GI (<10°) may also contribute to dampen this impact. Keeping the swing- or the stance-leg extended during the execution phase may therefore cause discomfort or pain to the swing knee, hip and above structures with task repetition, should the step length and progression velocity remain the same as in the control condition. In line, participants in the two orthosis conditions were able to reach the same step length as in the control condition when instructed to do so (cf. Complementary experiment), however they then reported joint discomfort. In congruence, the peak of Rz value immediately following the swing foot-contact was 6% larger in the orth-stance condition than in the control condition and, most importantly in terms of postural perturbation, the associated slope became much larger (21% and 25%, for the orth-swing and the orth-stance condition, respectively) than in the control condition. The perturbing effect of the vertical force impact was therefore increased by the orthosis wear. It thus seems likely that the reduced progression velocity and step length reported in the two orthosis conditions of the main experiment reflects a protective strategy directed to attenuate this vertical force impact and keep it the same as in the control condition.

A recent study by Honeine *et al*.^8^ showed that both hip abduction from the swing side and stance leg knee flexion is involved in the mediolateral shift of COP during APAs. In that study, the authors showed that, for small to shoulder-height stance width, the CNS relies principally on hip abduction. Stance-leg knee flexion, on the other hand, becomes predominant when starting gait from larger stance widths. It was still surprising that none of the spatiotemporal features of mediolateral APAs significantly changed when the orthosis was applied to the stance knee (orth-stance vs. control condition). It could be that the knee flexion required to laterally shift the COP during the APAs was too small (it is approximately 2–3° in the normal standing posture^8^) to be completely annihilated by the orthosis. Another plausible solution, is that the swing leg abductors have compensated for the stance knee hypomobility by increasing their activity. In fact, compensations of an altered joint functioning by changes in the activity of muscles crossing a non-perturbed joint have been reported in the literature^47–49^. This has been shown, for example, in a prolonged hopping task. In this task, fatigue predominantly occurs in the ankle extensor since these muscles are the main contributors to vertical propulsion in the hop. With fatigue, Bonnard *et al*.^47^ reported that young healthy participants landed with more flexed knees and with an increased activity in the biarticular *rectus femoris muscle* (non-altered muscles) indicating some compensation with fatigue between the knee and ankle joint. Two different strategies appeared to further compensate for the fatigue of the ankle extensor muscles: one was organized across joints and consisted in a heavier reliance of the knee extensor *vastus lateralis*, and the other was organized within the fatigued joint and consisted in an earlier preactivation of the *gastrocnemius*. In the same vein, Rios *et al*.^49^ reported that individuals with chronic ankle instability were able to compensate for their ankle deficits by using proximal musculature to maintain reduced postural sway while kicking a ball. Now, it is clear that one limitation of this comparison between the present experiment and these latter studies is the time allocated to participants to adapt to the joint perturbation, i.e. this time was shorter in the present study than in these latter studies. Given the design of the present experiment, it could not be investigated whether compensation across joints (ankle, knee and hip) also occurred in the course of the limited number of GI trials (n = 10) performed in the two orthosis conditions. Future study will investigate the possible progressive trial-to-trial changes in leg muscle activation and possible compensation across joint in response to knee hypomobility with intensive task repetition.

Results further showed that the duration of the execution phase was slightly greater (around 6%) in the orth-stance condition compared to the control condition. It seems that this small increase was not sufficient to induce a significant increase in the mediolateral COM fall at foot-contact (note however that the difference in the mean mediolateral COM velocity at this time between the control and the orth-stance condition almost reached significance, with p < 0.07). It was therefore surprising that the step width was significantly enlarged by 2 cm (8.5%) in this condition. This point is discussed in the paragraph below.

Recent lines of research have investigated the adaptability of the mediolateral APAs programming to various postural constraints during stepping-like tasks such as GI^5,6,29,50^, rapid thigh flexion^51–53^ or lateral leg flexion^27^. The postural constraints manipulated in these studies included the temporal pressure imposed on the task^5,6,51,53,54^, the presence of an obstacle to clear^5,6^, fear of falling induced by support surface height^27,55^, the instruction on the progression velocity^29^ and external load distribution^30,50^. In each of these situations, it was reported that the CNS systematically modulated the stabilizing features of the stepping tasks, including mediolateral APAs parameters and step width (leg stiffness was solely investigated in Yiou *et al*.^5^) so as to maintain the mediolateral MOS invariant. The relationship between these stabilizing features and the MOS was recently strengthened by mechanical modelling of the human body during GI^5^. The systematic invariance of the MOS when the postural constraints varied, led the authors to propose that this quantity would function as a stability control parameter that the CNS would set before task execution.

The present result that the MOS was different (greater) in the two orthosis conditions than in the control condition is thus original, markedly contrasting with these previous studies. In the two orthosis conditions, this larger MOS was due to the step width which was significantly larger than required to reach the same MOS as in the control condition. This step width enlarging ensured a greater stability by providing a larger mediolateral base of support size and therefore further minimized the risk of imbalance after swing foot-contact^11,12,29,44^. The present result thus suggests that in unusual situations with potential instability, such as the ones encountered by the participants in the two orthosis conditions, the CNS may use a more conservative strategy than in situations with more habitual postural constraints, such as those investigated in the studies reported above. Based on our previous studies and the present findings, it can be proposed that a reference MOS would be set by the CNS before stepping and this reference value would depend on an internal representation of the current state of the postural system, the task dynamics and the novelty of the task. The CNS would then plan the mediolateral APAs features and the step width so as to reach the desired reference MOS.

Now, it should be emphasized that the conservative strategy of step width enlargement adopted by participants in the orthosis conditions may not be optimal in term of muscle activation. Indeed, stepping from an enlarged step width requires a greater lower limb activation to propel the COM laterally towards the stance leg for the forthcoming step^8^. This strategy was a priori not necessary to maintain stability since all participants in the control condition used a smaller step width without experiencing any imbalance. As stressed above, it is noteworthy that participants were all able-bodied and performed only ten trials in each condition. The strategy of step width enlargement may thus probably reflect immediate adaption to the mechanical knee perturbation, and it was associated with task novelty. As the participants become more familiar with this perturbation, it is not excluded that this conservative strategy may progressively be abandoned and that step width then becomes the same as in the control condition.

In addition to step-width, another factor affecting stability during gait initiation is the vertical braking of the COM, which reduces the impact of the foot with the ground. The CNS seems to also anticipate global kinematics prior to step execution in order to ensure that the amplitude of the impact with the floor is comparable across condition regardless of swing- or stance-knee constraint. Results indeed showed that the peak of Rz value as well as its corresponding slope, reached the same value in the three experimental conditions. As argued above and shown in the complementary experiment, this invariance is possible because both step length and progression velocity reached lower values in the two orthosis conditions compared to the control condition. As for the existence of a putative reference MOS, it can be suggested that prior to step execution the CNS sets a reference value for vertical force impact that should not be exceeded at foot-contact. The step length and progression velocity would then be adjusted according to the mechanical constraints applied to the postural system so as to reach this reference value. Both step length and progression velocity are known to depend on the amplitude of anteroposterior APAs (in term of peak backward COP shift^1^). The reference value for the vertical force impact, and the associated discomfort or pain, would thus be factors taken into account in the planning of anteroposterior APAs. This statement is in line with recent studies which reported that bilateral high-level fatigue^56^ and experimentally-induced pain of tibialis anterior^57^ induced a protective strategy featured by anteroposterior APAs attenuation, with consequent smaller step length and progression velocity compared to the control condition (i.e. “no fatigue” and “no pain” condition). Similarly, by increasing the mediolateral stability and by maintaining the vertical force impact unchanged in the two orthosis condition, the CNS gave “priority” to body protection rather than to motor performance (cf. Yiou *et al*.^58^ for a review on this topic).

It is noteworthy that each GI-related output variable, i.e. the MOS, the vertical force impact and the associated step length and progression velocity, reached equivalent values in the two orthosis conditions. This result was striking because the biomechanical constraints were very different in these two conditions as the stance and swing-leg knee ensure different functions during GI as recalled in the paragraphs above. In continuity with the hypothesis proposed in the above paragraphs, this finding suggests that the centrally-set reference values for the MOS and the vertical impact force were the same in the two orthosis conditions. During locomotor activities, the same leg is alternately involved in swing and stance. Setting “global” reference values, i.e. values that can be applied bilaterally by the CNS, instead of setting “local” reference values, i.e. one for each leg independently, may therefore probably simplify the motor planning and ensure a more symmetrical forward body progression. Setting global reference values may explain why participants in the orth-stance condition enlarged the step width as they did in the orth-swing condition, while there was no a priori mechanical reason to do so in this condition. This statement is in line with previous studies focusing on the adaptation to unilateral change in lower limb mechanical properties induced by a mass addition around the leg segment of a single lower limb during human walking^58,59^. After the initial addition of the mass to the leg, asymmetrical walking resulted where the non-weighted leg was taking a longer step than the weighted leg; however, a more symmetrical walking pattern gradually developed. In the same vein, Donker *et al*.^60^ reported that when the right wrist or the right ankle was loaded, adaptations in the contralateral arm and leg musculature occurred so that the cadence was not affected. These studies showed that the adaptations to local perturbations were not restricted to the perturbed limb but induced a global reorganization of the locomotor activity. A similar phenomenon may explain that GI-related output variables remained equivalent between the two orthosis conditions despite the biomechanical constraints were different.

In conclusion, this study provides evidence that the application of a local mechanical constraint, herein induced by unilateral knee hypomobility, induces profound changes in the global organization of gait initiation, altering motor performance but ensuring greater stability. It was proposed that the CNS set “global” reference values for mediolateral stability (estimated with the MOS) and for the vertical disturbance at the time of swing foot collision with the ground (as estimated with the peak vertical force and the associated slope) before stepping. These reference values would be set based on an internal representation of the current state of the postural system, the task dynamics and its novelty. The CNS would then plan APAs features, step width and length so as to reach the desired reference values. Future studies should investigate the effects of wearing the orthosis by a patient population (for example, stroke patients). In addition, the results obtained in this study can help in developing a motorized knee joint. Such orthoses could use inertial measurement unit(s)^61^ to detect gait initiating and control the knee joint accordingly in order to enhance gait performance without affecting stability.

## Methods

### Participants

Two separate experiments were carried out with two different groups of participants. Nineteen healthy adults (10 men, aged 26 ± 4 years [mean ± SD], height 1.7 ± 0.1 m, body-mass 77 ± 13 kg) participated in the main experiment. Eight healthy adults (4 men, aged 32 ± 7 years, height 1.71 ± 0.07 m, body-mass 65.5 ± 10.4 kg) participated in a complementary study which was carried out with different participants than in the main experiment. All were free of any known neuromuscular disorders. All participants gave written informed consent after being instructed as to the nature and purpose of the experiment, which was approved by the local ethics committee of University Paris-Sud, Paris-Saclay (EA 4532). The study conformed to the standards set by the Declaration of Helsinki.

### Task and experimental procedure

#### Main experiment

Participants initially stood barefoot on a force-plate embedded at the beginning of a five-meter track. Feet were hip-width apart, arms were hanging loosely at either side of their body and gaze was directed toward a red cross marked on a wall 6 meters from the participants and positioned at eye level. The boundaries of the feet in the initial posture were outlined on the force-plate with chalk, and participants were instructed to systematically position their feet within these marks under the supervision of the experimenters.

**Figure 6:**
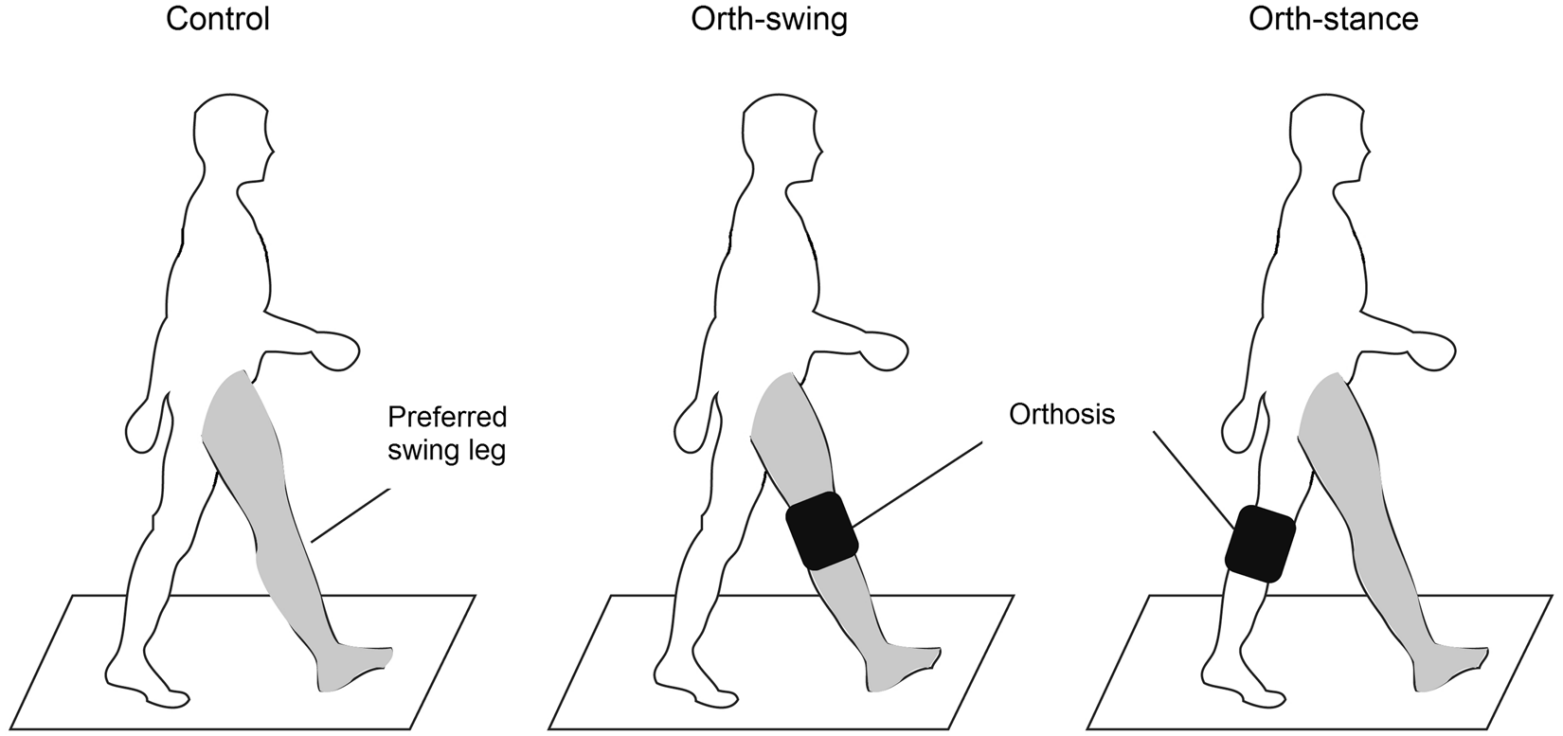
Illustration of the three experimental conditions. The orthosis was positioned over the knee of the stepping leg in the “orth-swing” condition and over the knee of the stance leg in the “orth-stance” condition.

Before recording, we determined the preferential starting foot of the subjects by applying a small thrust to their back forcing while standing eyes-closed resulting in a step forward. The procedure was repeated 3 times. Then, subjects were always instructed to initiate gait with the ‘preferred’ stepping leg that was determined by the test. Subjects were asked to repeat a trial when initiated with the ‘non-preferred’ leg. Participants performed three gait initiation conditions. Each condition was comprised of a series of ten gait initiation trials. The conditions were the following: 1) control condition: initiating gait without wearing the knee orthosis, 2) orth-swing condition: initiating gait with a knee orthosis over the swing-leg knee (i.e. the preferred stepping leg of participants) and 3) orth-stance condition: initiating gait with the orthosis over the stance-leg knee (i.e. the trailing leg). The experimental conditions are illustrated in Fig. 6. The order of the conditions was randomly assigned to participants to avoid rank effects, and a three- minute rest was imposed between two successive conditions to avoid fatigue. In each condition, participants were allowed two familiarization trials.

Participants initiated gait at a spontaneous velocity when they felt ready after receiving an “all set” signal; it was made clear that the “all set” signal was not a “go” signal and that they could take as much time as they needed to prepare their movements. The participants were repeatedly reminded of the task instructions. After initiating gait, participants walked to the end of the track, then stood still for a few seconds before returning to their starting position.

#### Complementary experiment

Results of the main experiment showed that participants spontaneously took a shorter step in the two orthosis conditions compared to the control condition. In contrast, the vertical force impact at the time of swing foot contact remained unchanged across the three conditions (cf. the paragraph “*Vertical force impact and vertical COM braking*” in the methods and the Results section). This result was surprising because it is known that the vertical force impact increases with step length, at least during running. This complementary experiment tested the hypothesis that participants shortened their step length in the two orthosis conditions in order to attenuate the vertical force impact, and thus, reduce joint discomfort or pain due to task repetition. This hypothesis implied that if participants were forced to attain the same step length in the two orthosis conditions as in the control condition, a higher vertical force impact than in the control condition would be reached. To test this hypothesis, the same protocol as in the main experiment was conducted, except that the step length was imposed, and was the same length as that spontaneously adopted in the control condition.

### Materials

**Figure 7:**
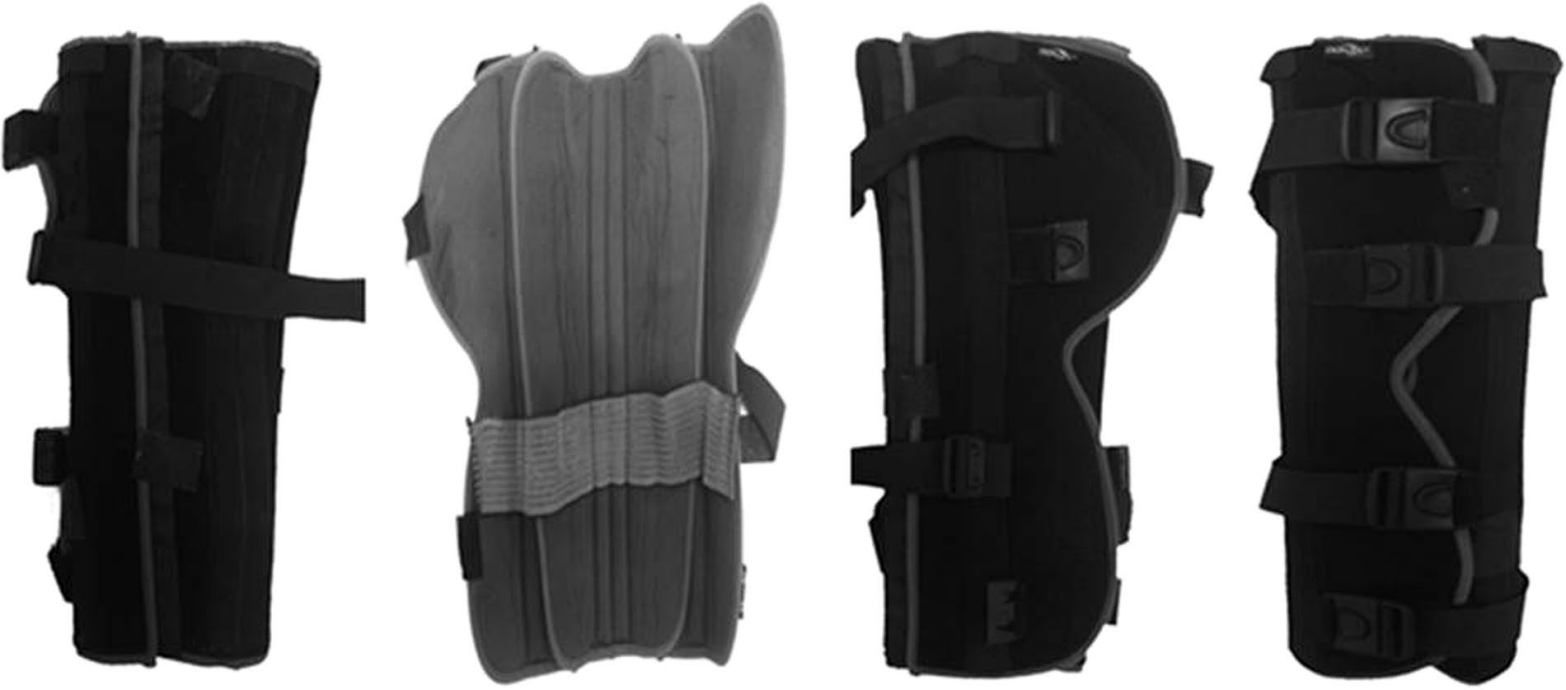
Postoperative knee orthosis (Donjoy®, referee AT43V).

Knee hypomobility refers to limiting flexion/extension at the knee to a maximum range of motion of 2° ^62^. This was achieved by the wearing of a postoperative knee orthosis (Donjoy®, referee AT43V) with a 52–57 cm height, two removable double bend posterior stays for immobilization of the knee, and inner elastic anti-migration strap (Fig. 7). This knee orthosis was adaptable for both the right or left knee.

External forces and moments applied to the participants were recorded from a force-plate (0.9 × 1.80 m, AMTI, Watertown, USA) that was large enough to allow the participant’s swing foot to systematically land on it at the end of GI. Force-plate data was recorded at an acquisition frequency of 500 Hz. Data acquisition and stimulus display were controlled by a custom-made program written in Matlab^TM^ (R2009b), The MathWorks (Inc., USA).

### Mechanical model

We used the same mechanical model as in a previous study to determine the stance leg stiffness along the mediolateral direction during the GI execution phase (for details on this model and related equations of motion, see Yiou *et al*.^5^). In brief, the human body was modeled as a single conic inverted pendulum which rotates around a fixed point corresponding to the stance ankle. It was considered that the COM was falling laterally under the influence of two forces: the gravity force **P** = m**g** (where m is the mass of the solid, and **g** is the gravitational acceleration) and an elastic restoring force **T** that reflects active muscular control of the movement^63,64^, with **T** = k|**yM**| where k is the stance leg stiffness and |**yM**| is the absolute value of the mediolateral COM shift, which was systematically oriented towards the swing leg (positive values) during the execution phase. The initial position and velocity of the cone corresponded to the position and velocity of the participant’s COM at toe off.

### Data reduction

Force-plate data were low-pass filtered using a Butterworth filter with a 15 Hz^65^ and a 10 Hz^29^ cut-off frequency, respectively. The mediolateral (yCOP) and anteroposterior (xCOP) coordinates of the COP were computed from force-plate data as follows:

1 $${\rm{yCOP}}=\frac{{\rm{Mx}}+{\rm{Fy}}\times {\rm{dz}}}{{\rm{Fz}}}$$

2 $${\rm{xCOP}}=\frac{-{\rm{My}}+{\rm{Fx}}\times {\rm{dz}}}{{\rm{Fz}}}$$

where Mx and My are the moments around the anteroposterior and mediolateral axes, respectively; Fy, Fx and Fz are the mediolateral, anteroposterior and vertical ground reaction forces; and *d*z is the distance between the surface of the force-plate and its origin.

Instantaneous COM acceleration along the anteroposterior and mediolateral axes was determined from the ground reaction forces (GRF) according to the Newton’s second law. COM velocity and displacement was computed by successive numerical integrations of COM acceleration using integration constants equal to zero, i.e. initial velocity and displacement null^1^.

### Dependant variables of the main experiment

#### Timing

The following instants were determined from biomechanical traces: gait initiation onset (t_0_), swing heel-off, swing toe-off, swing foot-contact and rear foot-off. These instants were determined from force-plate data^1,17,66^. Two t_0_ times were estimated, one for the mediolateral axis and one for the anteroposterior axis. The t_0_ times corresponded to the instants when the mediolateral or anteroposterior COP trace deviated 2.5 standard deviations from its baseline value.

#### Initial posture, APAs and foot lift phase

The mediolateral and anteroposterior COM position in the initial upright static posture was estimated by averaging the COP position during the 250 ms period preceding the “all set” signal. GI was divided into APAs (from t_0_ to heel-off), swing foot-lift (from heel-off to toe-off), and execution phase (from toe-off to foot-contact, Fig. 2). The duration of APAs along the mediolateral and anteroposterior axes were computed separately, because the t_0_ times for these two axes did not necessarily occur simultaneously^29^. The amplitude of APAs was characterized by the peaks of the backward and mediolateral COP shift obtained during the APAs time window. COM velocity and displacement along the mediolateral and anteroposterior axes were quantified at heel-off, toe-off and foot-contact. Because the statistical analyses revealed the same trend for significant differences across conditions for variables measured in both of the heel-off and toe-off instances, only results related to the COM kinematics at toe-off will be reported.

#### Execution phase

Spatiotemporal features of the GI execution phase included: execution phase duration, anteroposterior COM velocity at foot-contact (progression velocity), step length and stance leg stiffness. Step length corresponded to the difference between the most backward COP position during GI and the COP position at the time of rear foot-off^67^. Rear foot off time was marked by the onset of the second plateau of the mediolateral COP trace (Fig. 2). Finally, stance leg stiffness along the mediolateral direction was determined in each condition with the mechanical model described above.

#### Stability

An adaptation of the “margin of stability” (MOS) introduced by Hof *et al*.^68^ was used to quantify the mediolateral dynamic stability at foot-contact (hereafter referred to as “stability”). The MOS corresponded to the difference between the mediolateral boundary of the base of support (BOS_ymax_) and the mediolateral position of the “extrapolated COM” at swing foot-contact (YcoM_FC_). Thus:

3 $${\rm{MOS}}={{\rm{BOS}}}_{{\rm{ymax}}}-{{\rm{YcoM}}}_{{\rm{FC}}}$$

Participants systematically landed on the force-plate with the swing heel first, then the toe. Under such a foot landing strategy, it was shown that BOS_ymax_ could be estimated with the mediolateral COP position at the time of rear foot-off^30,67^. Based on the study by Hof *et al*.^68^ and the results from our previous studies^5,6,29,30^, the mediolateral position of the extrapolated COM at foot-contact (YcoM_FC_) was calculated as follows:

4 $${{\rm{YcoM}}}_{{\rm{FC}}}={{\rm{yCOM}}}_{{\rm{FC}}}+\frac{{\rm{y}}\text{'}{{\rm{COM}}}_{{\rm{FC}}}}{{\omega }_{0}}$$

where yCOM_FC_ and y’COM_FC_ are respectively the mediolateral COM position and velocity at foot-contact, and *ω*_0_ is the eigenfrequency of the body, modelled as an inverted pendulum and calculated as follows:

5 $${{\omega }}_{0}=\sqrt{\frac{g}{l}}$$

where *g* = 9.81 m/s^2^ is the gravitational acceleration and *l* is the length of the inverted pendulum, which in this study corresponded to 57.5% of body height^69,70^. Mediolateral dynamic stability at foot-contact is preserved on the condition that YcoM_FC_ is within BOS_ymax_, which corresponds to a positive MOS. A negative MOS indicates a mediolateral instability and implies that a corrective action is required to maintain balance, e.g. in the form of an additional lateral step.

Hence, variables related to the mediolateral stability included the MOS and its components, i.e. step width, mediolateral COM velocity and position at swing foot-contact. Step width corresponded to the difference between the most lateral position of the mediolateral COP trace obtained during the first plateau of the trace (Fig. 1) and the mediolateral COP position at the time of rear foot-off^67^.

#### Vertical force impact and vertical COM braking

The disturbing effect elicited by the collision of the swing foot with the ground was estimated with the peak and the slope of the vertical ground reaction force (Rz) trace reached immediately after the time of swing foot contact (Fig. 1). The slope was computed as the maximum of the time derivative of the Rz trace before the peak is reached. This slope has been proposed to be more representative of the disturbing effect than the peak value^68^. In addition, the capacity of participant to brake the vertical COM fall during the execution phase was estimated with the following index (braking index, BI), with BI = [vertical COM velocity at the time of swing foot-contact minus peak vertical COM velocity]/peak vertical COM velocity^2,71–76^.

### Dependant variables of the complementary experiment

In the complementary experiment, the step length was an independent variable. Only the vertical force impact variables and the vertical braking index (cf. paragraph above) were analysed.

### Statistics

Mean values and standard deviations were calculated for each variable in each condition. The normality of data was checked using the Kolmogorov-Smirnov test and the homogeneity of variances was checked using the Bartlett test. One way repeated measures (RM) ANOVAs with the condition (“control”, “orth-swing”, “orth-stance”) as within subject factor was used. A significant outcome was investigated with the Tukey *post hoc* test. The threshold of significance was set at p < 0.05.
